# Knockdown of nrf2 Exacerbates TNF-*α*-Induced Proliferation and Invasion of Rheumatoid Arthritis Fibroblast-Like Synoviocytes through Activating JNK Pathway

**DOI:** 10.1155/2020/6670464

**Published:** 2020-12-22

**Authors:** Yu Du, Qian Wang, Na Tian, Meng Lu, Xian-Long Zhang, Sheng-Ming Dai

**Affiliations:** ^1^Department of Rheumatology & Immunology, Shanghai Jiao Tong University Affiliated Sixth People's Hospital, Shanghai 200233, China; ^2^Department of Physiology and Pathophysiology, School of Basic Medical Sciences, Fudan University, Shanghai 200032, China; ^3^Department of Orthopedic Surgery, Shanghai Jiao Tong University Affiliated Sixth People's Hospital, Shanghai 200233, China

## Abstract

Fibroblast-like synoviocytes (FLS) in the synovial tissue of rheumatoid arthritis (RA) exhibit over-proliferative and aggressive phenotypes, which participate in the pathophysiology of RA. In RA, little is known about the nonantioxidant effect of nuclear factor erythroid 2-related factor 2 (nrf2), the master regulator of redox homeostasis. In this study, we aimed to explore the expression and upstream regulatory factors of nrf2 and revealed its functions in modulating the proliferation and invasion in RA-FLS. FLS were isolated from RA and osteoarthritis patients. Expression of nrf2 in the synovial tissues and FLS was analyzed by immunohistochemistry, real-time PCR, Western blotting, and immunofluorescence staining. Cell proliferation was examined by Cell Counting Kit-8. Cell invasion was tested by transwell assay. Phosphorylation of JNK was determined by Western blotting. The results showed that nrf2 expression in the RA synovial tissues was upregulated. TNF-*α* promoted expression and nuclear translocation of nrf2 in RA-FLS and increased the intracellular reactive oxygen species (ROS) level. Nrf2 nuclear translocation was blocked by ROS inhibitor N-acetylcysteine. Both knockdown of nrf2 by siRNA and inhibition of nrf2 by ML385 significantly promoted the TNF-*α*-induced proliferation and invasion of RA-FLS. Activation of nrf2 by sulforaphane (SFN) profoundly inhibited the TNF-*α*-induced proliferation and invasion of RA-FLS. Knockdown of nrf2 also enhanced the TNF-*α*-induced matrix metalloproteinases (MMPs) expression and phosphorylation of JNK in RA-FLS. Proliferation and invasion of RA-FLS incubated with TNF-*α* and nrf2 siRNA were inhibited by pretreatment with JNK inhibitor SP600125. In conclusion, nrf2 is overexpressed in synovial tissues of RA patients, which may be promoted by TNF-*α* and ROS levels. Activation of nrf2 may suppress TNF-*α*-induced proliferation, invasion, and MMPs expression in RA-FLS by inhibiting JNK activation, indicating that nrf2 plays a protective role in relieving the severity of synovitis in RA. Our results might provide novel insights into the treatment of RA.

## 1. Introduction

Rheumatoid arthritis (RA) is an autoimmune disease that primarily affects the joints, characterized by synovial inflammation and hyperplasia, autoantibody production, and cartilage and bone destruction, which would ultimately lead to irreversible functional loss [1]. Although major progress has been made in RA treatment, there are still concerns about incomplete responses and toxicity. Further elucidation of RA pathogenesis and the development of effective therapies remain an urgent need.

Synovium comprises an intimal lining layer and a sublining layer, where two major cell types are located: fibroblast-like synoviocytes (FLS) and macrophage-like synoviocytes. Normal synovium has a thin intimal lining layer consisting of two to three layers of cells; however, the intimal lining layer in RA becomes inflamed, hyperplastic with expansion in both cell types, and invasive of cartilage and bone [2, 3]. RA-FLS exhibits an aggressive tumor-like phenotype that increases the invasiveness into the extracellular matrix (ECM) and further aggravates the joint damage [4]. However, the mechanisms underlying the abnormal growth and invasion of RA-FLS are not fully understood.

Oxidative stress occurs in RA due to an imbalance in the generation and removal of reactive oxygen species (ROS), leading to inflammation, abnormal cellular behavior, and tissue damage [5]. Nuclear factor erythroid 2-related factor 2 (nrf2) as a transcription factor and a master regulator of antioxidant response has been considered a therapeutic target for RA [6]. Under basal conditions, nrf2 binds to the inhibitory Kelch-like ECH-associated protein 1 (Keap1) in the cytoplasm, which targets nrf2 to proteasomal degradation [7]. When being activated by ROS or electrophilic compounds such as sulforaphane (SFN), nrf2 translocates into the nucleus after being released from Keap1 and subsequently regulates the expression of targeted genes [8–10].

The transactivation of multiple genes involved in the antioxidant response, immune process, and metabolism by nrf2 has been well recognized [9]. It was originally considered to exert protection against oxidative stress and inflammation in various diseases including RA. Nrf2 deficiency aggravated disease severity and inflammatory condition in RA animal models [11, 12]. Chemicals that directly activate nrf2 showed anti-inflammatory and immunoregulatory effects [6, 13] Nrf2 also mediated the therapeutic effects of many drugs in RA, such as licochalcone A and resveratrol [14–16]. Nevertheless, recent studies noted that nrf2 could also play a role in cancer progression, promoting proliferation and invasion in glioblastoma and hepatocellular carcinoma [17–19]. These remind us that nrf2 might modulate RA disease severity through other mechanisms such as controlling cell growth and invasion. Upregulated nrf2 has been found in the FLS of RA patients, but how nrf2 modulates the proliferation and invasion of RA-FLS remains unknown [11].

Here, we confirmed the higher expression of nrf2 in the RA synovium and revealed that TNF-*α* contributed to the activation of nrf2 by inducing intracellular ROS in RA-FLS. More importantly, we identified knockdown of nrf2 as an enhancer of proliferation, invasion, and the expression of the matrix metalloproteinases (MMPs) of RA-FLS by activating the JNK pathway.

## 2. Materials and Methods

### 2.1. Tissue Samples

Synovial tissues from RA and osteoarthritis (OA) patients were obtained during the total knee arthroplasty. Patients were diagnosed according to the 2010 ACR/EULAR classification criteria for RA or 1986 revised ACR classification criteria for OA [20, 21]. This study complied with the Declaration of Helsinki (1964), and the research protocol was approved by the Ethic Committee of Shanghai Jiao Tong University Affiliated Sixth People's Hospital. Informed consent was obtained from all the patients for tissue donation.

### 2.2. Isolation and Culture of Human FLS

Synovial tissues were used to isolate primary FLS. The fat, fibrous membranes, and cartilage fragments were removed. The synovial tissue was minced into fragments and digested with 1 mg/mL type II collagenase (Merck Millipore, USA) dissolved in Dulbecco's Modified Eagle Medium (DMEM) at 37°C overnight. After that, the digested mixture was pipette through a 70 *μ*m mesh cell filter (Merck Millipore, USA) into a 50 mL tube and centrifuged at 250 g for 10 min. The supernatant was removed, and the cell pellet was washed with DMEM three times. Finally, the cell pellet was resuspended with DMEM supplemented with 10% fetal bovine serum (FBS) and 100 U/mL penicillin-streptomycin (Gibco, USA). The cells were cultured in 10 cm culture dishes at a density of 2.5 × 10^4^ cells/mm^2^ in a humidified incubator (Thermo Fisher Scientific, USA) containing 95% air and 5% CO_2_ at 37°C and subcultured upon confluence. Passages 3-8 were used for experiments.

### 2.3. Immunohistochemistry and Immunofluorescence Staining

Synovial tissues were fixed in 4% paraformaldehyde (PFA) for 48 h and embedded in paraffin. Serial sections (5 *μ*m) were made for immunohistochemistry and hematoxylin and eosin (HE) staining. For immunohistochemistry, synovial tissue sections were deparaffinized with xylene and rehydrated with gradient ethanol. Antigen retrieval was conducted with citrate buffer at 95°C for 8 min. For immunofluorescence staining, cells were fixed with 4% PFA for 10 min and permeabilized with 0.25% Triton X-100 in phosphate-buffered saline (PBS) for 10 min. Tissue sections or cells were blocked with 10% donkey serum (Jackson ImmunoResearch Labs, USA) in tris-buffered saline (TBS) for 1 h at room temperature and incubated with anti-nrf2 antibodies (Abcam, UK, 1: 500) or rabbit IgG (Merck Millipore, USA, 1 : 500) at 4°C overnight. Then, they were washed with TBS (0.025% Triton X-100) for 2 × 5 min. Next, tissue sections were incubated with horseradish peroxidase- (HRP-) conjugated donkey anti-rabbit IgG (Jackson ImmunoResearch Labs, USA, 1 : 1000) at room temperature for 1 h and stained with diaminobenzidine (DAB, Beyotime Biotechnology, China) to visualize the staining. Cells were incubated with Alexa Fluor 488-conjugated donkey anti-rabbit IgG secondary antibody (Abcam, UK, 1 : 200) in darkness for 30 min at room temperature. Cells were mounted with mounting media containing DAPI (4′,6-diamidino-2-phenylindole) (Beyotime Biotechnology, China). Digital images were captured from synovial tissue sections through an optical microscope (Olympus, Japan) and from cells with a fluorescence microscope (Olympus, Japan).

### 2.4. Western Blotting

Total protein of cultured RA-FLS was extracted using RIPA lysis buffer (Beyotime Biotechnology, China) on ice. Nuclear protein was extracted with Nuclear and Cytoplasmic Protein Extraction Kit (Beyotime Biotechnology, China) according to the manufacturer's instructions. The lysates were added by 5 × Gel Sample Loading Buffer (New Cell & molecular Biotech, China) and heated for 8 min at 100°C. The denatured samples were separated by 10% sodium dodecyl sulfate-polyacrylamide gel electrophoresis (SDS-PAGE) and transferred to polyvinylidene fluoride (PVDF) membranes (Merck Millipore, USA). After blocking with 5% bovine serum albumin (BSA) in TBS-Tween, the membranes were incubated overnight at 4°C with primary antibodies against nrf2 (Abcam, UK, 1 : 500), *β*-actin (Affinity, USA, 1 : 5000), Histone-3 (Cell Signaling Technology, USA, 1 : 1000), phospho-ERK (Cell Signaling Technology, USA, 1 : 1000), ERK (Cell Signaling Technology, USA, 1 : 1000), *β*-Tubulin (Affinity, USA, 1 : 5000), phospho-JNK (Cell Signaling Technology, USA, 1 : 1000), JNK (Cell Signaling Technology, USA, 1 : 1000), and GAPDH (Affinity, USA, 1 : 5000). After washing with TBS-Tween, membranes were incubated with HRP-conjugated goat anti-mouse (Affinity, USA, 1 : 5000) or anti-rabbit (Affinity, USA, 1 : 5000) antibodies, and protein bands were visualized using NcmECL Ultra enhanced chemiluminescent detection reagents (New Cell & Molecular Biotech, China).

### 2.5. RNA Extraction and Real-Time Quantitative PCR

Total RNA of cultured RA-FLS was extracted by Trizol reagents (Thermo Fisher Scientific, USA) and reverse-transcribed into cDNA using the ReverTra Ace qPCR RT Master Mix with gDNA Remover kit according to the manufacturer's instructions (Toyobo, Japan). Real-time PCR analysis was conducted using the SYBR Green Realtime PCR Master Mix kit (Toyobo, Japan) on the Quant Studios 7 Flex real-time PCR system (Applied Biosystems, USA). The primer sequences are listed in Table 1.

### 2.6. siRNA Transfection

RA-FLS were seeded in six-well plates and reached 80% confluence on the day of transfection. Cells were transfected with 70 nM nrf2 siRNA or scramble siRNA (Ribobio, China) by Lipofectamine RNAiMAX transfection reagent (Invitrogen, USA). Briefly, 7 *μ*L nrf2 siRNA (20 *μ*M) and an equal volume of transfection reagent were separately diluted in 125 *μ*L Opti-MEM I Medium (Gibco, USA). Diluted siRNA and diluted transfection reagent were mixed (1 : 1 ratio) and incubated for 5 min at room temperature. Then, the siRNA/Lipofectamine solution mixture was added to the cells.

### 2.7. ROS Detection

Intracellular ROS level was detected using DCFDA/H2DCFDA Cellular ROS Assay Kit (Abcam, UK) according to the manufacturer's protocols. In brief, cells were stained with 25 *μ*M 2,7-dichlorodi-hydrofluorescein diacetate (DCFDA) for 45 min at 37°C and stimulated with TNF-*α* (25 ng/mL) for 0.5, 1, 2, or 3 h, or tert-butyl hydroperoxide (TBHP) (50 *μ*M) for 3 h as the positive control. Signals were captured at 488 nm excitation wavelength with a fluorescence microscope (Olympus, Japan) at 200 magnification with equal exposure time.

### 2.8. Cell Proliferation Analysis

Cell proliferation assay was performed using the Enhanced Cell Counting Kit-8 (CCK-8) (Beyotime Biotechnology, China). RA-FLS were seeded in a 96-well plate at a density of 10^3^ cells per well and incubated with or without indicated reagents. Two or four days later, the cell culture medium was removed, and the diluted CCK-8 reagent was added to the cells. The optical density (OD) value at 450 nm was read on a microplate reader (Thermo Fisher Scientific, USA) after incubating for 2 h. The proliferation rate was expressed as the relative OD value at 450 nm.

### 2.9. Cell Invasion Analysis

The invasion assay was performed in a 24-well plate using 6.5 mm diameter inserts with membranes containing 8 *μ*m pores (BD Bioscience, USA). The inserts were coated with Corning Matrigel matrix (Corning, USA) at 300 *μ*g/mm^2^ according to the manufacturer's instructions. Cells were digested and seeded into the upper chamber of the transwell insert in 100 *μ*L DMEM without FBS at a density of 10^4^ cells/well. DMEM (650 *μ*L) containing 10% FBS was placed in the lower chamber. The transwell plate was incubated at 37°C for 2 days. Then, the chamber membrane was fixed with 4% PFA for 10 min and washed with double distilled water. Then the membrane was stained with crystal violet solution (Beyotime Biotechnology, China). Cells remaining on the upper surface of the chamber were wiped using a cotton swab. Pictures of the membrane were taken under an optical microscope (Olympus, Japan) at 200 magnification. The density of cells that migrated to the lower side of the membrane represented the cell invasion ability. The cell density for each assay was calculated as the mean density of 5 random fields.

### 2.10. Statistical Analysis

Data analysis was conducted using IBM SPSS Statistics 22.0, and graphs were plotted using GraphPad Prism 8 software. The statistical comparisons between two groups were calculated using Student's *t*-test. Multiple comparisons were calculated using one-way analysis of variance (ANOVA) or two-way ANOVA for repeated measurements with Dunnett's T3 post hoc test. *p* values < 0.05 were considered statistically significant.

## 3. Results

### 3.1. Upregulation of nrf2 in the Synovial Tissues of RA Patients

**Figure 1:**
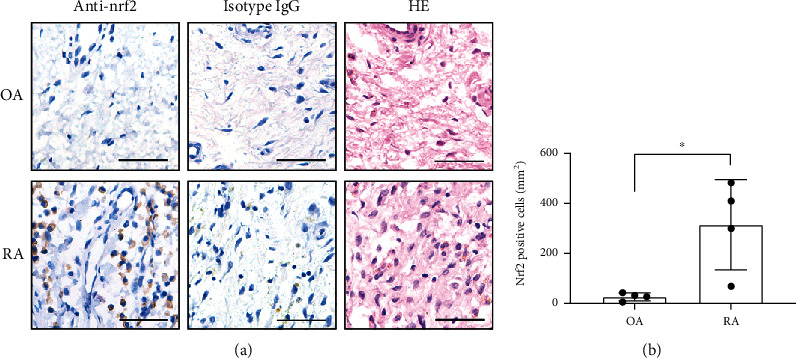
Nuclear factor erythroid 2-related factor 2 (nrf2) expression in rheumatoid arthritis (RA) and osteoarthritis (OA). (a) Immunohistochemistry staining using anti-nrf2 antibodies and rabbit IgG (isotype) and hematoxylin and eosin (HE) staining of RA and OA synovial tissues. (b) Nrf2-positive cell densities in the synovial tissues of RA and OA. Scale bars represented 50 *μ*m. *N* = 4. Data were shown as the mean ± standard deviation (SD); ^∗^*p* < 0.05.

Immunohistochemistry staining was performed to determine the nrf2 protein expression level in the synovial tissues from RA and OA patients using anti-nrf2 antibodies and rabbit IgG (isotype). Nrf2 expression was significantly upregulated in the RA synovium compared with OA; synovial hyperplasia was also observed in RA with HE staining (Figure 1).

### 3.2. Induction of nrf2 Expression and Nuclear Translocation by TNF-*α* through Inducing ROS

**Figure 2:**
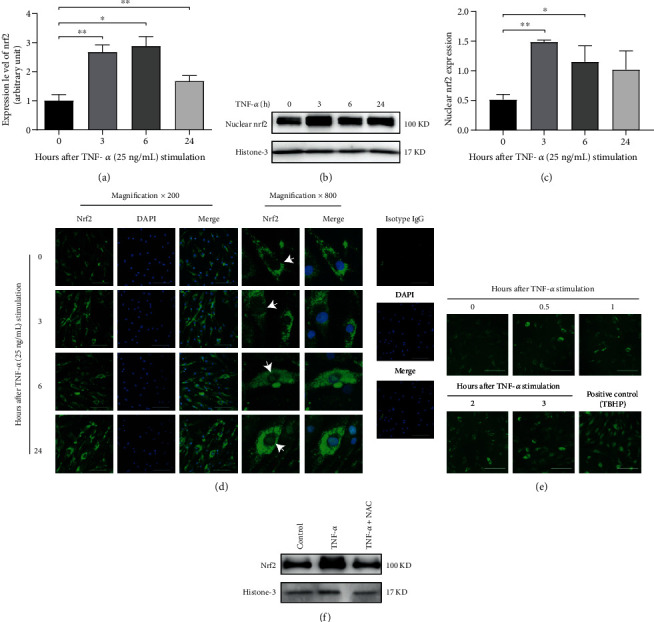
Induction of nrf2 expression and nuclear translocation by TNF-*α* through inducing ROS. The mRNA expression (a) and nuclear protein level (b, c) of nrf2 in RA-FLS were detected by real-time PCR and Western blotting after TNF-*α* (25 ng/mL) stimulation for 0, 3, 6, and 24 h. Histone-3 was used as the housekeeping gene in Western blotting. *N* = 3. (d) Nrf2 nuclear translocation (as shown by white arrows) after TNF-*α* (25 ng/mL) stimulation for 0, 3, 6, and 24 h was examined by immunofluorescence staining. Representative pictures (magnification ×200) from different groups and partially enlarged pictures (magnification ×800) are shown. Scale bar represented 50 *μ*m. (e) Intracellular ROS levels in RA-FLS stimulated with TNF-*α* (25 ng/mL) for 0, 0.5, 1, 2, and 3 h and tert-butyl hydroperoxide (TBHP, 50 *μ*M) (positive control) for 3 h were detected using DCFDA probe. Scale bar represented 100 *μ*m. (f) The nuclear protein level of nrf2 in RA-FLS after TNF-*α* (25 ng/mL) stimulation for 3 h with or without N-acetylcysteine (NAC, 5 *μ*M) pretreatment for 1 h was detected by Western blotting. Histone-3 was used as the housekeeping gene. Data were shown as the mean ± SD; ^∗^*p* < 0.05 and ^∗∗^*p* < 0.01.

TNF-*α* is one of the most important mediators of RA synovitis. To investigate whether TNF-*α* could cause nrf2 upregulation and activation, RA-FLS were treated with TNF-*α* (25 ng/mL). The upregulated expression of nrf2 mRNA was demonstrated after 3, 6, and 24 h of TNF-*α* treatment with real-time PCR analysis (Figure 2(a)). The nuclear protein level of nrf2 was increased in RA-FLS after stimulation with TNF-*α* (25 ng/mL) at different time points (Figures 2(b) and 2(c)), indicating that TNF-*α* could promote nrf2 nuclear translocation. Furthermore, the nuclear translocation of nrf2 after TNF-*α* (25 ng/mL) treatment was confirmed with immunofluorescence staining (Figure 2(d)).

To illustrate whether ROS mediates TNF-*α*-induced nrf2 activation, the intracellular ROS level of RA-FLS was detected. TBHP was used as a positive control. It was found that TNF-*α* (25 ng/mL) enhanced intracellular ROS levels in a time-dependent manner (Figure 2(e)). Further treatment with ROS inhibitor N-acetylcysteine (NAC) blocked TNF-*α*-induced nrf2 nuclear translocation (Figure 2(f)).

### 3.3. Enhancement of Proliferation and Invasion by Knockdown of nrf2 in RA-FLS

**Figure 3:**
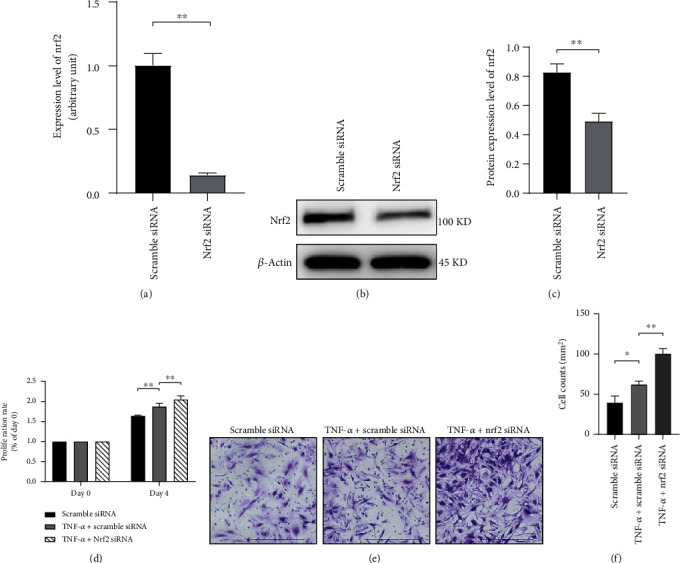
Knockdown of nrf2 promoted TNF-*α*-induced proliferation and invasion of RA-FLS. (a) Nrf2 mRNA expression was reduced by nrf2 siRNA transfection for 48 h, as revealed by real-time PCR. (b, c) The protein expression level of nrf2 was examined after nrf2 siRNA transfection for 72 h by Western blotting. *β*-Actin was used as the housekeeping gene. (d) The proliferation rate of RA-FLS was analyzed by CCK-8 assay after transfection with scramble siRNA or nrf2 siRNA for 72 h. Cells were examined before and after 4 days of culture in the presence or absence of TNF-*α* (25 ng/mL). The proliferation rate was expressed as the relative optical density (OD) value at 450 nm between day 4 and day 0. (e, f) Invasion of RA-FLS was analyzed with transwell assay after transfection with scramble siRNA or nrf2 siRNA for 72 h. Cells were cultured in the transwell chamber coated with Matrigel in the presence or absence of TNF-*α* (25 ng/mL). The density of cells that migrated to the lower side of the membrane represented the cell invasion ability, as visualized by crystal violet staining. Pictures were taken at 200 magnification. Scale bar represented 200 *μ*m. *N* = 3. Data were shown as the mean ± SD; ^∗^*p* < 0.05 and ^∗∗^*p* < 0.01.

Next, the biological effects of nrf2 in RA-FLS were explored. Successful nrf2 knockdown by siRNA was confirmed by real-time PCR and Western blotting (Figures 3(a)–3(c)). The effects of knockdown of nrf2 on the TNF-*α*-induced proliferation rate and invasion in RA-FLS were analyzed with CCK-8 and transwell assay. As a result, TNF-*α* (25 ng/mL) promoted the proliferation and invasion of RA-FLS, which were further enhanced by knockdown of nrf2 (Figures 3(d)–3(f)).

### 3.4. Effects of nrf2 Activator SFN and Inhibitor ML385 on Proliferation and Invasion of RA-FLS

**Figure 4:**
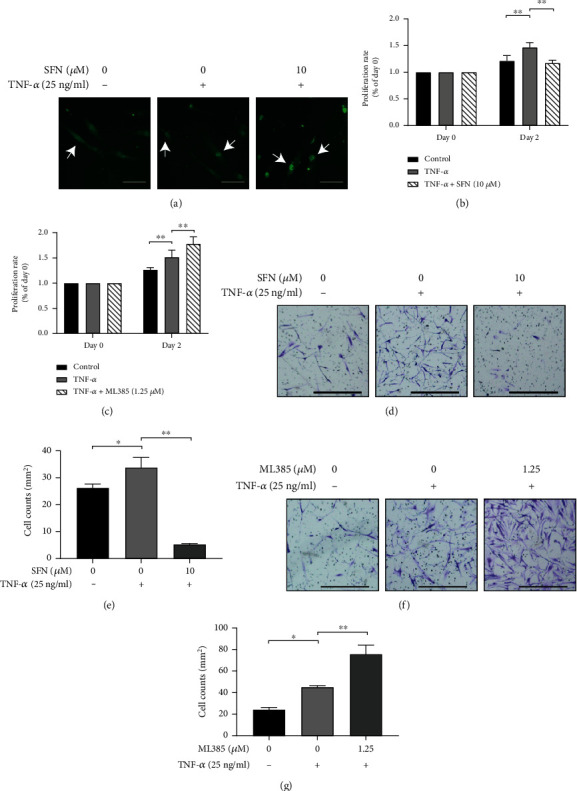
Effects of nrf2 activator and inhibitor on proliferation and invasion of RA-FLS. (a) Nrf2 nuclear translocation (as shown by white arrows) was tested with immunofluorescence staining in RA-FLS after pretreatment with nrf2 activator sulforaphane (SFN) at a concentration of 10 *μ*M for 1 h and subsequently stimulating with/without TNF-*α* (25 ng/mL) for 3 h. (b, c) Proliferation of RA-FLS treated with SFN or ML385 was analyzed by CCK-8 assay. Cells seeded in the 96-well plate were pretreated with SFN (10 *μ*M) (b) or ML385 (1.25 *μ*M) (c) for 1 h and subsequently stimulated with/without TNF-*α* (25 ng/mL) for 2 days. (d–g) Invasion analysis of RA-FLS treated by SFN or ML385 was performed with transwell assay. Cells seeded in the transwell chamber were pretreated with SFN (10 *μ*M) (d, e) or ML385 (1.25 *μ*M) (f, g) for 1 h and subsequently stimulated with/without TNF-*α* (25 ng/mL) for 2 days. Scale bar represented 200 *μ*m. *N* = 3. Data were shown as the mean ± SD; ^∗^*p* < 0.05 and ^∗∗^*p* < 0.01.

RA-FLS were incubated with nrf2 activator SFN or inhibitor ML385 in the presence or absence of TNF-*α* (25 ng/mL), to verify the influence of nrf2 on the TNF-*α*-induced proliferation and invasion of RA-FLS. SFN (10 *μ*M) exacerbated the TNF-*α*-induced nrf2 nuclear translocation (Figure 4(a)). Both proliferation and invasion of RA-FLS were inhibited by SFN (10 *μ*M) (Figures 4(b), 4(d), and 4(e)). ML385 is an nrf2 inhibitor that leads to repressed transcriptional activation function of nrf2 [22]. ML385 (1.25 *μ*M) profoundly accelerated TNF-*α*-induced invasion and proliferation of RA-FLS (Figures 4(c), 4(f), and 4(g)).

### 3.5. Promotion of the TNF-*α*-Induced MMP Expression by Knockdown of nrf2

**Figure 5:**
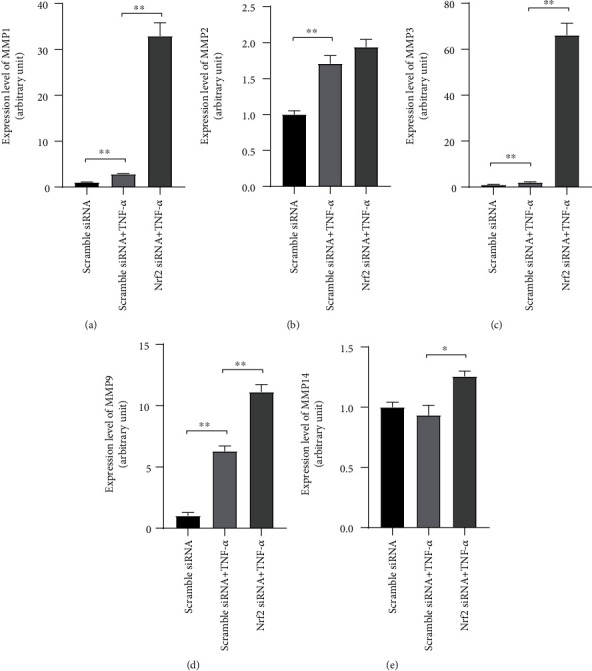
Knockdown of nrf2 promoted TNF-*α*-induced matrix metalloproteinase (MMP) expression. After transfection with scramble siRNA or nrf2 siRNA for 3 days, RA-FLS were stimulated with/without TNF-*α* (25 ng/mL) for 24 h. The expression of MMP1 (a), MMP2 (b), MMP3 (c), MMP9 (d), and MMP14 (e) was examined with real-time PCR. *N* = 3. Data were shown as the mean ± SD; ^∗^*p* < 0.05 and ^∗∗^*p* < 0.01.

Proteins of the MMP family degrade the extracellular matrix and mediate cell invasion. The mRNA expression of MMP1, MMP2, MMP3, MMP9, and MMP14 in RA-FLS after treatment of TNF-*α* (25 ng/mL) and knockdown of nrf2 were analyzed with real-time PCR. TNF-*α* (25 ng/mL) upregulated the expression of MMP1, MMP2, MMP3, and MMP9 in RA-FLS, and knockdown of nrf2 further enhanced the TNF-*α*-induced expression of MMP1, MMP3, and MMP9, without affecting the expression of MMP2 (Figures 5(a)–5(d)). TNF-*α* (25 ng/mL) did not influence the expression of MMP14, but knockdown of nrf2 would promote the MMP14 expression (Figure 5(e)).

### 3.6. JNK Mediating the Modulation of Proliferation, Invasion, and MMP9 Expression in RA-FLS by Knockdown of nrf2

**Figure 6:**
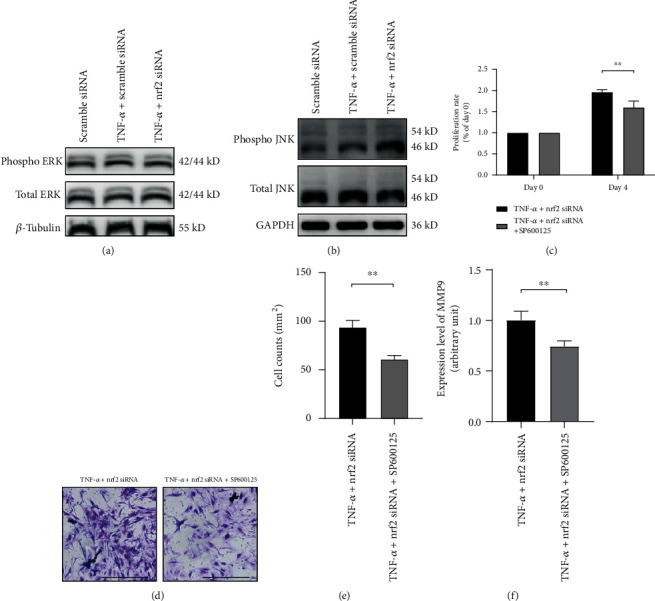
JNK pathway mediates the modulation of proliferation, invasion, and MMP9 expression in RA-FLS by nrf2. (a) After transfection with scramble siRNA or nrf2 siRNA for 3 days, RA-FLS were further stimulated with/without TNF-*α* (25 ng/mL) for 24 h. Representative figures show the phosphorylation of ERK examined by Western blotting. *β*-Tubulin was used as the housekeeping gene. (b) Representative figures show the phosphorylation of JNK examined by Western blotting. GAPDH was used as the housekeeping gene. (c) After transfection with nrf2 siRNA for 3 days, the proliferation of RA-FLS was analyzed by CCK-8 assay. Cells were cultured in the 96-well plate and stimulated with TNF-*α* (25 ng/mL) in the presence or absence of SP600125 (20 *μ*M) for 4 days. (d, e) After transfection with nrf2 siRNA for 3 days, invasion of RA-FLS was analyzed with transwell assay. Cells were cultured in the transwell chamber and stimulated with TNF-*α* (25 ng/mL) in the presence or absence of SP600125 (20 *μ*M) for 2 days. (f) After transfection with nrf2 siRNA for 3 days, RA-FLS were further stimulated with TNF-*α* (25 ng/mL) in the presence or absence of SP600125 (20 *μ*M) for 24 h. The MMP9 mRNA expression was examined by real-time PCR. Scale bar represented 200 *μ*m. *N* = 3. Data were shown as the mean ± SD; ^∗∗^*p* < 0.01.

To elucidate whether MAPK pathways mediate the enhanced proliferation, invasion, and MMP expression by knockdown of nrf2 in RA-FLS, phosphorylation of ERK and JNK was examined.Neither TNF-*α* (25 ng/mL) treatment nor nrf2 siRNA changed the phosphorylation level of ERK (Figure 6(a)). The phosphorylation of JNK was upregulated by TNF-*α* (25 ng/mL) treatment, and knockdown of nrf2 facilitated the TNF-*α*-induced JNK phosphorylation (Figure 6(b)). JNK pathway inhibitor SP600125 was applied to block the phosphorylation of JNK, and SP600125 (20 *μ*M) was found to be effective in remarkably suppressing the proliferation and invasion of RA-FLS incubated with TNF-*α* and nrf2 siRNA (Figures 6(c)–6(e)). The mRNA expression of MMP9 induced by knockdown of nrf2 and TNF-*α* (25 ng/mL) was also significantly inhibited by SP600125 treatment (Figure 6(f)). The mRNA expression levels of MMP1, MMP2, MMP3, and MMP14 were not changed (data not shown).

## 4. Discussion

The present study demonstrated that TNF-*α* promoted the nrf2 expression and nuclear translocation by upregulating intracellular ROS level in RA-FLS. Silencing nrf2 further facilitated the TNF-*α*-induced RA-FLS proliferation and invasion by promoting the expression of MMPs partly through activating the JNK pathway. These results indicate that nrf2 acts as a negative-feedback regulator in the RA-FLS against TNF-*α*-induced proliferation, invasion, and MMP expression. It plays a protective role in relieving the severity of synovitis in RA.

First, nrf2 protein expression was verified in the human synovial tissues. Nrf2 expression in RA synovial tissues was much higher than OA, which was consistent with the previous study where Wruck et al. [11] demonstrated that nrf2 was activated in RA synovium compared with healthy donors. Proinflammatory cytokines, such as TNF-*α*, IL-1, and IL-6, play pivotal roles in the pathogenesis of RA. TNF-*α* is one of the most established effectors that mediate synovial inflammation and imbalanced systemic immune status [23]. In monocytes, TNF-*α* induced sustained nrf2 activation and increased expression of nrf2 targeted genes [24]. Besides, a low concentration of TNF-*α* evoked significant nuclear translocation of nrf2 and transactivation of nrf2 targets [25]. Here, it was found that TNF-*α* not only upregulated nrf2 expression but also triggered nrf2 nuclear translocation, indicating that increased TNF-*α* level in RA patients might at least partly account for the nrf2 overexpression in the RA synovium. Then, the mechanisms that mediated the TNF-*α*-induced nrf2 activation were explored. It has been well recognized that intracellular ROS and electrophiles are potent nrf2 activators by modulating the conformation of the nrf2 adaptor keap1 and stop the nrf2 from going through keap1-dependent ubiquitination and proteasomal degradation [9]. TNF-*α* could increase ROS production in mouse embryonic fibroblasts, Hela cells, and murine hepatocytes [26–28]. As expected, in the present study, TNF-*α* induced ROS production in RA-FLS, and pretreatment with ROS scavenger NAC inhibited the TNF-*α*-induced nrf2 nuclear translocation, indicating that ROS mediated the TNF-*α*-induced nrf2 activation in RA-FLS.

Despite the widely studied powerful antioxidant ability, nrf2 exerts multiple nonantioxidant effects. Recent studies addressed that nrf2 modulated cell proliferation, survival, and invasion, although the modulating direction depended on different cellular microenvironments. Nrf2 deficiency disrupted self-renewal of the airway basal stem cells [29]. Knockout of nrf2 delayed proliferation of hepatocytes after hepatectomy [30]. Nevertheless, type 2 innate lymphoid cells from nrf2-deficient mice underwent hyperproliferation in response to the stimulation of IL-33 combined with IL-2, IL-7, or thymic stromal lymphoprotein [31]. ML385 is a probe molecule that binds to the Neh1 domain of nrf2 and interferes with the binding of V-maf avian musculoaponeurotic fibrosarcoma oncogene homologue G with nrf2 and blocks the nrf2 targeted genes expression [22]. Here, both knockdown of nrf2 by siRNA and inhibiting nrf2 by ML385 significantly promoted the TNF-*α*-induced proliferation of RA-FLS. SFN is the bioactive form of glucoraphanin that is mostly found in cruciferous vegetables; it effectively activates nrf2 by modifying the sensor cysteine in keap1 [32]. The present study further demonstrated that activating nrf2 by SFN profoundly inhibited the TNF-*α*-induced proliferation of RA-FLS. All these data suggest that increased and activated nrf2 secondary to inflammatory microenvironment may counteract the hypertrophy of RA synovium.

The invasive characteristic of RA-FLS is one of the major causes of bone and cartilage destruction in RA. So far, how nrf2 modulates the invasion of RA-FLS has not been elucidated. Most studies regarding the effect of nrf2 on cell invasion were conducted in cancer cells, and the results were paradoxical. Overexpression of nrf2 in oral squamous cell carcinoma cells promoted cell migration and invasion [33]. However, stabilizing nrf2 in breast cancer cells inhibited invasion and expression of recepteur d'origine nantais, a tyrosine kinase receptor that promotes cancer cell invasion [34]. The present study showed that both silencing nrf2 and inhibiting nrf2 by ML385 promoted the invasion of RA-FLS. On the contrary, activating nrf2 by SFN inhibited the TNF-*α*-induced invasion of RA-FLS. These results further suggest that nrf2, being a negative feedback regulator against TNF-*α*-induced synovitis in RA, might exert a protective effect by downregulating the invasiveness of RA-FLS.

Rheumatoid synoviocytes express a battery of MMPs that are the primary effectors responsible for pathologic ECM remodeling and cell invasion in RA [35]. The expression levels of MMP1, MMP2, MMP3, MMP9, and MMP14 were tested, which are among the major types contributing to the process of joint destruction in RA [36, 37]. It was found that knockdown of nrf2 could enhance the expression of these MMPs. Consistent with our results, Choi et al. revealed that nrf2 activator SFN inhibited IL-1*β*-induced expression of MMP-1, MMP-3 in RA-FLS [38]. Activation of nrf2 reduced UVA-mediated MMP1 upregulation in human keratinocyte cell line [39]. Moreover, disruption of nrf2 enhanced the MMP9 expression after spinal cord injury in mice [40]. In summary, the above data suggest that nrf2 may downregulate the invasiveness of RA-FLS by inhibition of MMP production.

The promoter regions of many MMPs contain the binding site of c-Fos/activator protein-1 (AP-1), which is critical for MMPs expression and locates downstream of the JNK MAPK and ERK MAPK kinases [41]. It has been well recognized that TNF-*α* regulates the expression of MMPs via the MAPK signaling cascades in RA-FLS [42]. Knockdown of nrf2 could also activate the MAPK pathways in UV-radiated human keratinocytes [39, 43]. Thus, the pathways involving JNK and ERK were analyzed in the present study. As a result, the phosphorylation of JNK was enhanced in the RA-FLS treated with TNF-*α* alone, and the upregulated phosphorylation of JNK was further enhanced by nrf2 siRNA. Besides, blocking JNK with its inhibitor SP600125 reduced not only the invasion but also the MMP9 expression mediated by knockdown of nrf2 in RA-FLS. In addition, several studies noted the proproliferative effect of JNK pathway [44, 45], which was in line with the finding that the proliferation of RA-FLS incubated with TNF-*α* and nrf2 siRNA was inhibited by pretreatment with JNK inhibitor SP600125. These data suggest that nrf2 may inhibit the activation of JNK pathway to downregulate MMP expression, invasion, and proliferation of RA-FLS.

## 5. Conclusions

In conclusion, nrf2 is overexpressed in synovial tissues of RA patients, which may be promoted by TNF-*α* and ROS levels. Increased nrf2 may suppress TNF-*α*-induced proliferation, invasion, and MMPs expression in RA-FLS through inhibiting JNK activation. Our findings offer a novel insight into the protective role that nrf2 played in RA.

## Figures and Tables

**Table 1:** Primer sequences for real-time PCR.

Gene symbol	Forward primer (5′-3′)	Reverse primer (5′-3′)
nrf2	TCAGCGACGGAAAGAGTATGA	CCACTGGTTTCTGACTGGATGT
MMP1	TCAGGGGAGATCATCGGGAC	GTCCAAGAGAATGGCCGAGT
MMP2	GATACCCCTTTGACGGTAAGGA	CCTTCTCCCAAGGTCCATAGC
MMP3	ACTGGAGATTTGATGAGAAGAGAA	TGGGTCAAACTCCAACTGTGA
MMP9	AGACCTGGGCAGATTCCAAAC	CGGCAAGTCTTCCGAGTAGT
MMP14	CGAGGTGCCCTATGCCTAC	CTCGGCAGAGTCAAAGTGG
*β*-Actin	GGACCTGACTGACTACCTCAT	CGTAGCACAGCTTCTCCTTAAT

## Data Availability

All data are available from the corresponding author on reasonable request.
